# User involvement in service delivery predicts outcomes of assistive technology use: A cross-sectional study in Bangladesh

**DOI:** 10.1186/1472-6963-12-330

**Published:** 2012-09-20

**Authors:** Johan Borg, Stig Larsson, Per-Olof Östergren, ASM Atiqur Rahman, Nazmul Bari, AHM Noman Khan

**Affiliations:** 1 Department of Health Sciences, Lund University, Malmö, Sweden; 2 Social Medicine and Global Health, Lund University, Malmö, Sweden; 3 Institute of Social Welfare and Research, University of Dhaka, Dhaka, Bangladesh; 4 Centre for Disability in Development, Savar, Bangladesh

## Abstract

**Background:**

Knowledge about the relation between user involvement in the provision of assistive technology and outcomes of assistive technology use is a prerequisite for the development of efficient service delivery strategies. However, current knowledge is limited, particularly from low-income countries where affordability is an issue. The objective was therefore to explore the relation between outcomes of assistive technology use and user involvement in the service delivery process in Bangladesh.

**Methods:**

Using structured interviews, data from 136 users of hearing aids and 149 users of manual wheelchairs were collected. Outcomes were measured using the International Outcome Inventory for Hearing Aids (IOI-HA), which was adapted for wheelchair users. Predictors of user involvement included preference, measurement and training.

**Results:**

Users reported outcomes comparable to those found in other high- and low-income countries. User involvement increased the likelihood for reporting better outcomes except for measurement among hearing aid users.

**Conclusions:**

The findings support the provision of assistive technology as a strategy to improve the participation of people with disabilities in society. They also support current policies and guidelines for user-involvement in the service delivery process. Simplified strategies for provision of hearing aids may be explored.

## Background

Are there any benefits of involving users in the process of delivering assistive technology services? Excluding user-oriented elements in the process may help cut costs, and thereby make assistive technology more affordable, particularly in less-resourced settings where access to assistive technology is limited and affordability is a major issue [1].

Little is known about outcomes of assistive technology use in developing countries. Available studies from low- and lower-middle-income countries indicate that the use of hearing aids is beneficial for language development and education, and is effective in enhancing participation, that the use of leg prosthesis facilitate mobility, work and sports, and that the use of manual wheelchairs has a positive impact on health, quality of life, and activities and participation [2-5]. To our knowledge, nothing has been published on how the outcomes in these countries relate to elements of the service delivery process.

Experts have recommended that users in low-income countries should be involved in the delivery of services, that individual assessments should be made, and that users should receive relevant training in order to benefit from using assistive technology [6,7]. This user-centered approach is favored in well-resourced countries where the concern – besides positive outcomes – is not so much whether people will be able to access assistive technology or not, but whether they will use or abandon the products once they have got them [8-11]. A recent study in the USA found a significant relationship between feeling informed and satisfaction with assistive technology [12]. The same study also found that feeling that personal needs were not assessed led to lower satisfaction and that lack of user involvement in the decision making process resulted in somewhat greater rates of abandonment.

To support development of cost-efficient strategies for provision of assistive technology, the extent to which people benefit from using assistive technology and the factors that may impact the benefits need to be identified. The objective of this study was therefore to explore the relation between self-reported outcomes of hearing aid and manual wheelchair use among people with disabilities in Bangladesh and their involvement in the delivery of assistive technology services.

## Methods

### Context

This study was undertaken in Bangladesh, which has an estimated population of about 164 million people living on 147 thousand square kilometers of land. In 2009, the country ranked 146 on the Human Development Index. The life expectancy was 65.7 years and the adult literacy rate was 53.5%. About two out of five lived below the national poverty line and about one out of two lived on less than $1.25 a day [13,14].

The disability prevalence rate in Bangladesh is about 6% [15], which corresponds to approximately 10 million people. Reportedly, disability has a devastating effect on quality of life, particularly on educational attainment and employment [16]. Bangladesh adopted the Persons with Disability Welfare Act in 2001, which was followed by the ratification of the CRPD in 2007 and its Optional Protocol in 2008. Although the country supports equal rights and opportunities for people with disabilities in principle, for most of them these rights have not been realized as their access to development programmes, social benefits, and health and rehabilitation services is limited [17,18].

According to an estimate of the World Health Organization (WHO), about 1.6 million people in Bangladesh would need a wheelchair [6]. Considering the situation in countries like Indonesia and Nigeria, it can be assumed that about five million or more Bangladeshis would benefit from using a hearing aid [7]. Despite government, non-government and private initiatives to make assistive technology accessible, the needs for assistive technology are far from being met [19,20]. Users or their families usually pay the full or a subsidized price. Occasionally, wheelchairs are distributed in relatively large volumes at various ceremonies [20]. Apart from services being economically, geographically and physically inaccessible, lack of trained personnel is another reason for this gap [21].

### Sample

The sample included in this study was derived from a cross-sectional survey, using an interviewer-administered structured questionnaire to collect quantitative data. It aimed at exploring the relationship between the use of assistive technology and the enjoyment of human rights and the economic situation of men and women with hearing and ambulatory impairments of 15 to 55 years of age living in urban and rural Bangladesh. Survey data had been collected from 285 people with hearing impairment and 298 people with ambulatory impairment. All invited potential respondents agreed to participate.

Due to a lack of government registers of people with disabilities, the non-government organization Centre for Disability in Development (CDD) was contacted in order to find eligible respondents. CDD is the largest disability oriented, national resource and training centre in Bangladesh. Through its more than 300 partner organizations across the country, CDD has access to locally maintained registers of people with disabilities, including users of assistive technology. People had been included in the registers in various ways. Main methods to identify people with disabilities had been: community meetings attended by people with disabilities, information provided by community people, home visits based on information from local people and authorities, people with disabilities voluntarily approaching the organizations, people with disabilities referring other people with disabilities, and surveys. The proportion of people being recruited by what method is unknown.

In the survey, representation from four typical areas of Bangladesh was sought; in and around the capital Dhaka, general countryside, areas prone to flooding, and hilly areas. In order to minimizing the number of involved organizations in the selected areas while still achieving a reasonable sample size, eight organizations were selected for collection of data from people with ambulatory impairments and ten organizations were selected for collection of data from people with hearing impairments in eight districts (Bogra, Chittagong, Dhaka, Gaibandha, Jhenaidah, Lalmonirhat, Meherpur, and Savar). The sample was recruited by eight and ten interviewers, respectively. In all sampling areas, all registered and eligible users of hearing aids and wheelchairs were included.

The population of interest in this study are men and women using hearing aids or wheelchairs aged 15–55 years. Those of the original survey that used hearing aids or wheelchairs were therefore included. This resulted in a sample of 136 users of hearing aids (62.5% men, mean age 26.5, age range 15–55, 64.7% living in villages) and 149 users of manual wheelchairs (73.8% men, median age 31.8, age range 15–55, 71.1% living in villages). To achieve a statistical power of 0.8 when using logistic regression and an effect size corresponding to an odds ratio (OR) of 2.0, a sample size of 141 is required. (See Additional file 1 for calculation details.) Users of hearing aids had used such devices for a mean time of 5.7 years (SD = 4.2) and users of wheelchairs had used them for a mean time of 4.5 years (SD = 3.7).

### Instrumentation

The questionnaire used for collecting data included a part on demographics and a part on provision and outcomes of assistive technology. Outcomes of hearing aid use were measured by the International Outcome Inventory for Hearing Aids (IOI-HA) [22,23], and outcomes of wheelchair use were measured by an adapted version of the IOI-HA, where ‘hearing aid’ had been replaced by ‘wheelchair’, and ‘hear’ had been replaced by ‘move around’. The reason for adapting the IOI-HA to wheelchair users was that it addresses areas considered relevant to wheelchair users too. However, it should be noted that although the wheelchair adapted IOI-HA has high face validity, the IOI-HA was developed for hearing aid users. As the range of issues influencing the IOI-HA outcomes may vary between different types of assistive technology, immediate conclusions cannot be drawn by comparing the outcomes between hearing aids and wheelchairs. In this study, the Cronbach alpha coefficients were 0.84 for hearing aids and 0.77 for wheelchairs, which indicate good internal consistency of the IOI-HA for both types of assistive technology.

### Procedure

Developed in English, the questionnaire was translated into Bangla. It was reviewed by native and non-native speakers of Bangla, including an expert on communication in simple Bangla, and revised. The questionnaire was then pre-tested on 30 people representing various respondent groups, which was followed by a minor revision.

An instruction manual for interviewers was developed. Ten interviewers, who worked with rehabilitation of people with disabilities in their respective organization, were recruited. They participated in a four-day training on interviewing and data collection techniques, which included a day of practice interviewing using the questionnaire. Following input from the training, the questionnaire was finalized. The interviewers were supervised by a coordinator and collected data between 6 November 2009 and 1 February 2010. Verbal, questionnaire-based interviews were conducted at the respondent’s home at a single occasion. In order to protect confidentiality of data, family members and neighbours were requested to provide privacy. In interviews where the interviewer was unable to communicate with a participant, data was collected through or from a proxy; most often the mother, but also the father, a sibling or another relative or person.

### Ethical considerations

As there is no authority in Bangladesh that grants ethical approvals, the University of Dhaka was consulted. Their ethical research praxis was followed, which meant that potential participants were informed about the study and invited to participate. Only those giving verbal consent were included in the study. Due to a high rate of illiteracy, written informed consent could not be used. Respondents could refuse to answer any of the questions or discontinue the interview at any time. Incentives for participation were not offered.

### Outcome variables

The outcome variables, with the lower and upper end of their respective 5-point response scale in brackets, were:

1. *Use* – indicates duration of daily use (‘None’ to ‘More than 8 hours’).

2. *Improved activity* – indicates how much the assistive technology has helped (‘Not at all’ to ‘Very much’).

3. *Residual activity limitation* – indicates how much difficulty remains (‘Very much’ to ‘No’).

4. *Satisfaction* – indicates whether the assistive technology is worth the trouble (‘Not at all’ to ‘Very much’).

5. *Residual participation restrictions* – indicates how much the hearing or moving difficulties have affected the things the user can do while using assistive technology (‘Very much’ to ‘Not at all’).

6. *Impact on others* – indicates how much the user thinks others were bothered by his or her hearing or moving difficulties while using assistive technology (‘Very much’ to ‘Not at all’).

7. *Quality of life* – indicates how much the assistive technology has changed the enjoyment of life (‘Worse’ to ‘Very much better’).

The response for each variable was transformed into a score within a range of 1–5, where higher scores signify more favorable outcomes.

### Predictor variables

User involvement in the service delivery was measured using the predictor variables preference, measurement, and training. *Preference* was measured by the Yes or No responses to the questions:

1. Did anyone at the facility ask you what type of hearing aid/wheelchair you need or want?

2. Did anyone at the facility ask you where you want to use the hearing aid/wheelchair?

3. Did anyone at the facility ask you for what purpose you want to use the hearing aid/wheelchair?

Among hearing aid users, *Measurement* was measured by the Yes or No responses to the questions:

1.Did anyone at the facility measure your hearing BEFORE you got the hearing aid?

2. Did anyone at the facility measure your hearing AFTER you got the hearing aid?

And among wheelchair users, *Measurement* was measured by the Yes or No response to the question:

1. Did anyone at the facility take any measurements of your body before you got the wheelchair?

*Training* was measured by the Yes or No responses to the questions:

1. Did you receive any training on how to use the hearing aid/wheelchair?

2. Did you or anyone in your family receive any training on how to maintain the hearing aid/wheelchair?

Wheelchair users were also asked:

3. Did you receive any training on how to prevent pressure sores?

*Preference* was given the value ‘Asked’ if the respondent had been asked at least one of the three questions; otherwise the value was set to ‘Not asked’. *Measurement* was given the value ‘Measured’ if the respondent had been measured at least once; otherwise the value was set to ‘Not measured’. *Training* was given the value ‘Trained’ if the respondent had received at least one type of training; otherwise the value was set to ‘Not trained’.

### Potential confounding variables

The outcomes were analyzed with respect to possible confounding variables, including *Place of living*, *Sex* and *Age*. To determine *Place of living*, the two categories ‘village’ and ‘town/city’ were used.

### Analyses

Questionnaire responses were recorded in a Microsoft Access database and analyzed using Statistical Package for Social Sciences (SPSS) version 17.0 statistical software. Descriptive statistics were used to report on differences between respondent groups. Crude odds ratios and 95% confidence intervals (95% CI) were calculated to explore associations between predictor and outcome variables, which were dichotomized. Analysis by logistic regression was performed to investigate the potential importance of possible confounders and to analyze whether service delivery elements can predict differences in outcomes.

The outcome variables were dichotomized to allow for logistic regression. The points of dichotomization varied, see Table 1, and were chosen in order to reduce the risk for overfitting by maximizing the number of respondents in the smaller of the two categories. Data on *Quality of life* was missing for two respondents using hearing aids. Among wheelchair users, data on *Residual activity limitations* was missing for one respondent, data on *Residual participation restrictions* was missing for one respondent, and data on *Impact on others* was missing for two respondents.

**Table 1:** Dichotomization points and number of participants in each category

**Domains**	**Hearing aid users**		**Wheelchair users**	
	**Score**	**n**	**Score**	**n**
*Use*				
Shorter daily use	1-3	43	1-3	58
Longer daily use	4,5	93	4,5	91
*Improved activity*				
Less improved activity	1-3	42	1-3	38
More improved activity	4,5	94	4,5	111
*Residual activity limitations*				
More activity limitations	1-4	74	1-3	46
Less activity limitations	5	62	4,5	102
*Satisfaction*				
Less satisfied	1-4	86	1-4	103
More satisfied	5	50	5	46
*Residual participation restrictions*				
More participation restrictions	1-4	56	1-4	70
Less participation restrictions	5	80	5	78
*Impact on others*				
More impact on others	1-4	24	1-4	48
Less impact on others	5	112	5	99
*Quality of life*				
Less improved quality of life	1-4	80	1-4	93
More improved quality of life	5	54	5	56

To further minimize the risk for overfitting, i.e. less than 10–15 events per predictor and confounding variable [24], not all potential confounding variables were included in the models for all outcomes. When not all potential confounding variables were included, those were selected whose odds ratios were statistically significant and/or reduced the adjusted odds ratio among the predictor variables the most. Among hearing aid users, adjusted logistic regression analysis was not possible to carry out regarding *Impact on others*.

A complementary analysis of the difference in self-reported listening capacity and listening performance between hearing aid users who had their hearing measured (n = 108) and those who had not had their hearing measured (n = 28) was carried out using t-tests. Listening capacity and listening performance were measured according to the International Classification of Functioning, Disability and Health (ICF) [25] using a 5-point Likert-type scale ranging from ‘Unable’ to “No difficulty’ and “Complete problem’ to “No problem’, respectively. Capacity excluded hearing aid use while performance included hearing aid use.

In addition, logistic regression was carried out to investigate the association between satisfaction and wheelchair users being asked all three preference questions (n = 55) versus being asked none, one or two of the questions (n = 93).

## Results

The numbers of positive responses to the questions about preference, measurement and training are presented in Table 2. Among the hearing aid users, 71% had been asked at least one question, while 60% of the wheelchair users had been asked one or more questions. Having been asked about purpose was the most frequent preference question among both hearing aid users (65%) and wheelchair users (58%). Seventy-nine percent of the hearing aid users and 34% of the wheelchair users had been measured at least once. Training had been received at least once by 53% of the hearing aid users and 41% of the wheelchair users. Among hearing aid users, training on maintenance (46%) was more common than training on use (39%). And among wheelchair users, training on use (29%) and training on maintenance (29%) were equally common. About 8% of the wheelchair users had received training on prevention of pressure sores.

**Table 2:** Number of positive responses to questions about preferences, measurements and training

**Predictor variables**	**Hearing aid users**		**Wheelchair users**	
	**n**	**%**	**n**	**%**
Asked at least one question	96	70.6	89	60.1
* Asked type of assistive technology needed/wanted*	*69*	*50.7*	*59*	*39.6*
* Asked where assistive technology will be used*	*76*	*55.9*	*69*	*46.6*
* Asked purpose of using assistive technology*	*89*	*65.4*	*86*	*57.7*
Measured at least once	108	79.4	50	33.6
* Measured before getting assistive technology*	*105*	*77.2*	*50*	*33.6*
* Measured after getting assistive technology*	*72*	*52.9*	*-*	*-*
Received at least one training	72	52.9	61	40.9
* Training on use of assistive technology*	*53*	*39.0*	*43*	*28.9*
* Training on maintenance of assistive technology*	*62*	*45.6*	*43*	*28.9*
* Training on pressure sore prevention*	*-*	*-*	*12*	*8.1*

Mean outcome scores from this study are presented in Table 3 along with reported mean outcome scores of hearing aid use from five other countries [4,26-28]. Apart from *Residual activity limitations*, the mean outcome scores of hearing aid users and wheelchair users are similar. Compared to the outcome scores in the other five countries, the scores for hearing aid users in Bangladesh are among the higher.

**Table 3:** Outcome scores in Bangladesh and outcome scores for hearing aid users in five other countries

**Domains**	**Hearing aid users**	**Wheelchair users**	**Hong Kong**	**Nether-lands**	**Nigeria**	**USA**	**Wales**
	**Mean (SD)**	**Mean (SD)**	**Mean**	**Mean**	**Mean**	**Mean**	**Mean**
Use	3.70 (1.10)	3.79 (0.89)	3.26	4.34	4.1	3.73	3.7
Improved activity	3.75 (1.20)	3.95 (0.79)	3.53	3.19	3.5	3.39	4.1
Residual activity limitations	4.21 (0.88)	3.81 (0.85)	4.42	3.51	3.4	3.40	4.2
Satisfaction	4.20 (0.76)	4.14 (0.74)	3.32	3.61	3.7	3.20	4.4
Residual participation restrictions	4.38 (0.89)	4.30 (0.89)	4.21	3.71	3.5	3.57	4.0
Impact on others	4.78 (0.57)	4.58 (0.69)	4.68	3.84	3.4	3.79	4.2
Quality of life	4.26 (0.74)	4.28 (0.66)	3.32	3.25	3.8	3.19	4.2

Crude odds ratios for studied outcomes for users who were asked about their preferences, measured and trained during the service delivery process compared to those who were not are presented in Table 4. Among both hearing aid users and wheelchair users there were statistically significant associations between predictor and outcome variables for *Less activity limitations*, *More satisfaction*, *Less participation restrictions* and *More improved quality of life*. Among hearing aid users, there was also a statistically significant association for *More improved activity*.

**Table 4:** Crude odds ratios (95% CI)

	**Outcomes**						
	**Longer daily use**	**More improved activity**	**Less activity limitations**	**More satisfaction**	**Less participation restrictions**	**Less impact on others**	**More improved quality of life**
Hearing aid users							
Asked	0.78 (0.58-1.04)	0.80 (0.60-1.07)	**1.53** (1.16-2.02)	**1.48** (1.11-1.98)	1.12 (0.86-1.46)	0.84 (0.59-1.19)	**1.37** (1.04-1.80)
Measured	1.00 (0.63-1.58)	1.36 (0.86-2.14)	1.43 (0.92-2.21)	1.44 (0.91-2.28)	1.21 (0.78-1.86)	0.72 (0.40-1.31)	1.46 (0.93-2.30)
Trained	1.34 (0.87-2.04)	**1.65** (1.06-2.57)	0.95 (0.64-1.39)	1.32 (0.88-1.97)	**2.56** (1.63-4.01)	0.89 (0.54-1.48)	0.73 (0.49-1.10)
Wheelchair users							
Asked	0.92 (0.47-1.81)	1.52 (0.72-3.19)	**3.06** (1.49-6.30)	1.37 (0.66-2.82)	**2.25** (1.15-4.40)	1.25 (0.62-2.51)	**2.19** (1.08-4.46)
Measured	1.20 (0.60-2.44)	0.96 (0.44-2.09)	1.44 (0.68-3.08)	**2.82** (1.36-5.82)	1.22 (0.62-2.42)	0.91 (0.44-1.88)	1.93 (0.96-3.88)
Trained	0.86 (0.44-1.69)	2.01 (0.91-4.45)	**2.62** (1.22-5.62)	**5.03** (2.38-10.6)	**2.18** (1.11-4.27)	1.40 (0.69-2.86)	**2.94** (1.48-5.85)

The adjusted odds ratios modeled by composite predictor variables of preference, measurement and training after adjustment for *Place of living*, *Age* and/or *Sex* are presented in Table 5.

**Table 5:** Adjusted odds ratios (95% CI)

	**Outcomes**						
	**Longer daily use**	**More improved activity**	**Less activity limitations**	**More satisfaction**	**Less participation restrictions**	**Less impact on others**	**More improved quality of life**
Hearing aid users							
Asked	0.58 (0.23-1.49)	0.46 (0.17-1.24)	**2.96** (1.20-7.34)	1.67 (0.69-4.05)	2.20 (0.85-5.72)	-	1.77 (0.73-4.30)
Measured	1.14 (0.42-3.11)	1.01 (0.36-2.85)	0.99 (0.37-2.66)	1.63 (0.58-4.60)	0.87 (0.30-2.50)	-	1.55 (0.56-4.34)
Trained	1.53 (0.71-3.31)	**2.66** (1.20-5.91)	0.68 (0.32-1.44)	1.66 (0.79-3.50)	**3.72** (1.68-8.22)	-	0.52 (0.24-1.09)
Living in village	**0.36** (0.15-0.87)	**0.40** (0.16-0.97)	**0.39** (0.18-0.86)	1.29 (0.59-2.84)	**0.35** (0.15-0.82)	-	0.68 (0.31-1.47)
Age	-	-	1.03 (0.996-1.06)	0.99 (0.96-1.02)	**1.06** (1.02-1.09)	-	1.00 (0.97-1.03)
Male	-	-	1.06 (0.50-2.29)	-	-	-	-
Wheelchair users							
Asked	0.86 (0.39-1.90)	1.27 (0.52-3.07)	**2.46** (1.04-5.82)	**0.36** (0.13-0.99)	2.06 (0.79-5.38)	1.27 (0.54-3.02)	1.36 (0.59-3.12)
Measured	1.52 (0.67-3.47)	0.76 (0.30-1.92)	0.93 (0.36-2.37)	**3.91** (1.50-10.2)	0.82 (0.31-2.18)	0.64 (0.26-1.58)	1.46 (0.64-3.32)
Trained	0.84 (0.39-1.81)	2.28 (0.94-5.57)	**2.47** (1.02-5.94)	**7.79** (3.00-20.2)	**4.27** (1.63-11.2)	1.80 (0.77-4.25)	**2.55** (1.18-5.51)
Living in village	0.60 (0.27-1.34)	0.52 (0.21-1.29)	**0.27** (0.10-0.70)	**0.22** (0.08-0.57)	**0.092** (0.03-0.27)	-	0.66 (0.29-1.50)
Age	1.02 (0.99-1.04)	-	-	-	**0.94** (0.91-0.97)	**0.95** (0.92-0.98)	-
Male	1.73 (0.80-3.75)	-	-	-	**8.63** (3.04-24.5)	**2.46** (1.05-5.80)	**0.43** (0.20-0.94)

Hearing aid users who had been asked about their preferences were more likely to report less activity limitations, OR = 3.0 (1.2-7.3). Measuring hearing was not associated with any statistically significant differences in outcomes. Having received training increased the likelihood for more improved activity, OR = 2.7 (1.2-5.9), and less participation restrictions, OR = 3.7 (1.7-8.2). Living in a village reduced the likelihood for longer daily use, OR = 0.36 (0.15-0.87), more improved activity, OR = 0.40 (0.16-0.97), less activity limitations, OR = 0.39 (0.18-0.86), and less participation restrictions, OR = 0.35 (0.15-0.82). Increased age by one year increased the likelihood for less participation restrictions, OR = 1.06 (1.02-1.1).

Wheelchair users who had been asked about their preferences were more likely to report less activity limitations, OR = 2.5 (1.0-5.8), and less satisfaction, OR = 0.36 (0.13-0.99). Having been measured increased the likelihood for reporting more satisfaction, OR = 3.91 (1.5-10). Training increased the likelihood for reporting less activity limitations, OR = 2.5 (1.02-5.94), more satisfaction, OR = 7.8 (3.0-20), less participation restrictions, OR = 4.3 (OR = 1.6-11), and improvements in quality of life, OR = 2.6 (1.2-5.5). Living in a village reduced the likelihood for less activity limitations, OR = 0.27 (0.10-0.70), more satisfaction, OR = 0.22 (0.08-0.57), and less participation restrictions, OR = 0.092 (0.03-0.27). Older age reduced the likelihood for less participation restrictions, OR = 0.94 (0.91-0.97), and less impact on others, OR = 0.95 (0.92-0.98). Men were more likely to report less participation restrictions, OR = 8.6 (3.0-24), and less impact on others, OR = 2.5 (1.05-5.8), while they were less likely to report improvements in quality of life, OR = 0.43 (0.20-0.94).

The analysis of self-reported listening capacity and listening performance did not reveal any statistically significant differences between hearing aid users who had had their hearing measured and those who had not (p = 0.446 and p = 0.366, respectively).

After adjusting for place of living, measurement and training, the odds ratio for reporting more satisfaction among wheelchair users being asked all questions versus not being asked all questions was 0.42 (0.17-1.08).

## Discussion

In order to explore the relation between outcomes of assistive technology use and user involvement in the service delivery process in a low-income country, cross-sectional data from users of hearing aids and manual wheelchairs in Bangladesh was analyzed using logistic regression. The results of this study indicate that the outcomes of hearing aid and wheelchair use are comparable with those reported in other high- and low-income countries. Statistically significant associations were found between the outcomes of using hearing aids and wheelchairs and the way these products were provided. The positive relation between training and outcomes is supported by previous studies [29].

The Convention on the Rights of Persons with Disabilities (CRPD) requires States to ensure access to affordable assistive technology and stresses the individual’s freedom to make own choices [30], which is to be respected in the provision of assistive technology as well [31]. In addition to the moral and legal support expressed in the CRPD, the findings offer empirical support for a user-centered delivery of assistive technology services, which is promoted in current guidelines.

Due to the temporal order between service delivery, assistive technology use and the outcome measurements, it may be argued that the studied relations are causal.

Compared to wheelchair users, participants using hearing aids were more involved in the service delivery, particularly regarding measurement.

### Hearing aid outcomes

Compared to hearing aid users in other countries, the users in Bangladesh seem to benefit equally well or better from their hearing aids [4,26-28]. Asking users about their preferences and providing them with training on use or maintenance of their products are indicative of more improved activity, less activity limitations and less participation restrictions. It is therefore a matter of concern that only two thirds of the hearing aid users had been asked preference related questions and about half of them had received any training. The findings indicate that user preferences and training need to be considered in the delivery of hearing aid services.

Contrary to our expectations, those who had had their hearing measured during the assessment process did not report better outcomes than those who did not have their hearing measured. One reason for this may be that the measuring of hearing was not carried out satisfactorily or did not have an effect on the selection and setting of the hearing aid. Another reason may be that the hearing measurement did not add any benefits among a majority of the participants, which the result of the analysis of listening capacity and performance indicate. As the mean outcome scores were relatively high, this result could indicate that all potential users of hearing aids may not need to have their hearing measured by an audiometer to benefit from using hearing aids. A delivery strategy that allows for a manual screening test while maintaining safety could potentially reduce the cost for providing hearing aids, and thereby make them more widely available in less-resourced settings. Potential users who require further assessments, or do not find themselves benefitting from the simple service, would be referred to appropriately resourced hearing centers. However, before low-cost service delivery methods are developed, which attempt to meet the needs for hearing aids among those with very basic needs without the use of expensive or inaccessible resources, further studies are required.

Users living in villages were less likely to report better outcomes. The reason for this is not known, but it might be that full participation in a town or city requires better hearing than in a village, and thus the benefits from using a hearing aid in a town or city are even more appreciated.

### Wheelchair outcomes

The findings support the current recommendations for user involvement in the provision of manual wheelchairs by the WHO [6]. Using an adapted version of the IOI-HA to score outcomes of wheelchair use is novel, although adaptations of the IOI-HA have been suggested to include non-hearing-aid-based interventions for people with hearing impairments [23]. The outcome scores are similar to those reported by hearing aid users across six of the seven domains. The higher level of residual activity limitations reported by hearing aid users may be explained by the physical environment being less accessible to wheelchairs, which is indicated by the adjusted odds ratios for wheelchair users living in villages compared to those living in towns or cities.

The findings in this study offer support for the importance of measuring the user and, particularly, providing training in use, maintenance and/or pressure sore prevention in order to achieve high outcomes. Two thirds of the respondents had not been measured, nearly three quarters had not been trained on wheelchair use or trained on wheelchair maintenance, and more than nine tenths had not been trained on pressure sore prevention. Lack of delivery of appropriate wheelchair services may result in debility, danger and death of users [32]. Inappropriate or poorly fitted wheelchairs have been reported to contribute to deaths [33], and discomfort has been found to be a cause of rejection of wheelchairs [34]. Thus, there is a need to raise awareness about the importance of including proper assessment, fitting and training in the delivery of wheelchair services.

Asking about a user’s preferences is associated with less activity limitations. On the contrary, asking about preferences is indicative of less satisfaction. The reason for this is unknown to the authors, but an explanation might be that asking about preferences might have given rise to expectations or requirements which could not be met with available types of wheelchairs.

### Limitations

The study has several limitations which should be considered when interpreting the findings. An inherent limitation of a cross-sectional design is its inapplicability in exploring cause and effect relationships. Although there is a temporal difference between delivery of assistive technology services and the collection of outcome data, which is indicative of a causal relation, longitudinal studies are needed to assess the causality. As only current users of assistive technology were included, we do not know to what extent elements of the service delivery process affect abandonment of assistive technology.

Like most countries, Bangladesh does not maintain a national register of users of assistive technology. As the prevalence of assistive technology use is low, it was impossible to achieve a representative sample considering the resource constraints of this study. In low-income countries, it is often difficult to obtain representative samples, particularly when hidden and vulnerable population groups are involved [35,36]. As the sample in this study was not randomly selected, there is a risk for selection bias. We must therefore be cautious about generalizing the findings to all users of hearing aids and wheelchairs in the population of interest. In addition, as indicated by the power calculation, the sample of hearing aid users would preferably have been a little larger. Lack of statistical power may result in an inability to identify associations.

It is possible that the respondents did not correctly recall what questions they had been asked, whether they had been measured or what training they had received resulting in misclassification. By using composite predictor variables, we have tried to minimize the chance of reporting no involvement when in fact the respondent had been asked a question, had been measured or had received training. Assuming a positive association between involvement and outcomes, this approach would be conservative and potentially underestimate the strengths of the studied associations, while a negative association could be overestimated. This assumption is supported by the result of the analysis among wheelchair users of the relation between satisfaction and asking all three questions versus asking none, one or two questions, which yielded a reduction in the strength of the negative relation as well as an indication that it is not statistically significant.

As all potential respondents participated and data was missing for less than 2% of the participants for each studied association, it is unlikely that the findings are biased by non-response or missing data.

The use of an administered questionnaire with scales can result in systematically biased answers. Although responses may be given to satisfy the interviewer, respondents usually have a tendency to avoid ends of scales. In addition, the understanding of Likert-type scales may vary, which can influence individual responses. As we only compare data provided by respondents within this single country context, these possible biases may not significantly affect the conclusions. Another limitation is that we relied on self- and proxy-reported data and do not know how closely the responses correlate with objective measures.

To avoid overfitting, all potential confounders were not accounted for in all studied associations. Despite this limitation, the results indicate that place of living, age and sex were significantly associated with the outcomes. Thus, demographic factors need to be considered in the delivery of assistive technology services.

It is not known from this study how measurements were taken or how the training was provided. Questions like these need to be addressed in future research if our understanding of intervention related factors influencing use is to be expanded [29].

Besides self-rated capacity and performance among respondents, the study did not take into account the severity, type (e.g., unilateral or bilateral) and duration of their impairments.

Although reliability in terms of internal consistency was good, the adapted version of the IOI-HA for wheelchair users has not been validated. A simple instrument that can be used for assessing outcomes of a wide range of assistive technologies would be useful. Based on the apparently high face validity of the IOI-HA and the experiences from using it in this study, it is suggested that studies are undertaken to explore its validity for other types of assistive technology than hearing aids.

## Conclusions

The findings support the provision of assistive technology as a strategy to improve the participation of people with disabilities in society. They also support current policies and guidelines for user-involvement in the provision of assistive technology in low-income countries. The lack of association between the outcomes and hearing measurement calls for further investigations. If it is confirmed, simplified services for provision of hearing aids may be explored.

## Competing interests

JB, SL, POÖ and ASMAR declare that they have no competing interests. NB and AHMNK work in a non-governmental organization whose activities include provision of assistive technology. However, they have not been involved in the analysis and interpretation of data.

## Authors’ contributions

JB conceived of the study, participated in its design, performed the statistical analysis, and drafted the manuscript. SL conceived of the study, participated in its design, and helped to draft the manuscript. POÖ participated in the design of the study, helped to perform the statistical analysis, and helped to draft the manuscript. ASMAR participated in the design of the study, helped in acquisition of data, and helped to draft the manuscript. NB participated in the design of the study, supervised acquisition of data, and helped to draft the manuscript. AHMNK participated in the design of the study, helped in acquisition of data, and helped to draft the manuscript. All authors read and approved the final manuscript.

## Pre-publication history

The pre-publication history for this paper can be accessed here:

http://www.biomedcentral.com/1472-6963/12/330/prepub

## Supplementary Material

Additional file 1 Power protocol. Click here for file
