# Supplementary material for: User involvement in service delivery predicts outcomes of assistive technology use: A cross-sectional study in Bangladesh
Source: BMC Health Serv Res. 2012 Sep 20;12:330. doi: 10.1186/1472-6963-12-330 (PMC3511287; doi:10.1186/1472-6963-12-330)
Supplement: Additional file 1 — Power protocol. [file 1472-6963-12-330-S1.doc]

**Appendix 1. Power protocol**

The power calculation was performed using G*Power 3.1.2 with the input parameter values indicated below.

**z tests -** Logistic regression

**Options:** Large sample z-Test, Demidenko (2007) with var corr

**Analysis:** A priori: Compute required sample size

**Input:** Tail(s) = Two

Odds ratio = 2

Pr(Y=1|X=1) H0 = 0.2

α err prob = 0.05

Power (1-β err prob) = 0.8

R² other X = 0.2

X distribution = Normal

X parm μ = 0

X parm σ = 1

**Output:** Critical z = 1.9599640

Total sample size = 141

Actual power = 0.8020939
